# Expanding the Applicability of Iliac Branch Devices in East Asian Patients

**DOI:** 10.1016/j.jacasi.2026.01.038

**Published:** 2026-05-02

**Authors:** Jose I. Torrealba, Tilo Kölbel

**Affiliations:** German Aortic Center Hamburg, Department of Vascular Medicine, University Heart Center, Hamburg, Germany

**Keywords:** abdominal aortic aneurysm, common iliac artery aneurysm, computational fluid dynamics, endovascular aortic repair, iliac branch device

**Figure:**
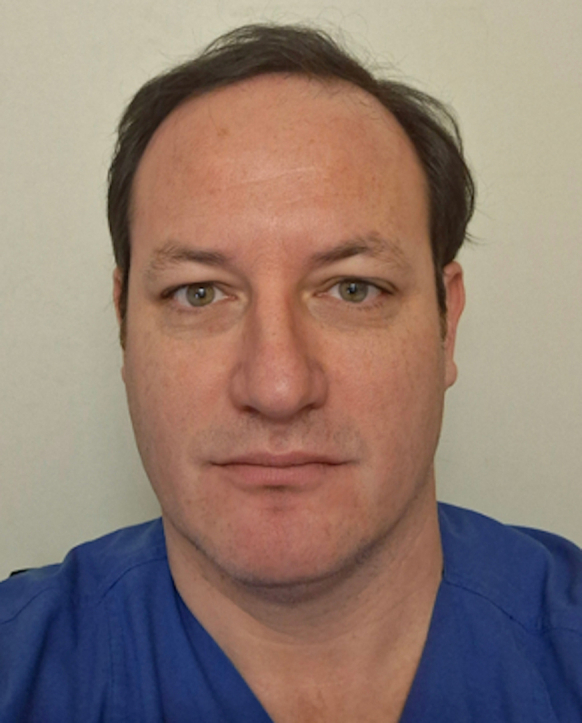

More than 15 years ago, iliac branch devices (IBDs) were introduced and have consistently demonstrated excellent outcomes, with long-term patency rates exceeding 90% and low reintervention rates,^1^ making them an ideal option for hypogastric artery (HA) preservation in endovascular aortoiliac repair. However, most of the available evidence regarding current devices and their instructions for use (IFUs) is derived predominantly from studies conducted in Western populations. Consequently, the real anatomical suitability of commercially available IBDs in other populations—particularly in East Asian patients—is limited, with fewer than 25% of patients meeting IFU criteria, as reported by Wu et al.^2^ Morphological limitations of the suitability of endovascular aneurysm repair and especially IBDs in East Asian populations are a shorter length and a smaller diameter of the common iliac artery (CIA), limiting contralateral access to the antegrade IBD branch and not providing sufficient room for an outer branch to open fully. To deal with the limited CIA length, a double-bifurcated stent-graft design using a 1-way valve and a graft fenestration for contralateral access has been proposed but after an initial successful case series, nothing further has been reported.^3^

To solve the issue of smaller CIA diameters, Wu et al^4^ described a physician-modified endograft for the iliac bifurcation, incorporating an inner branch sewn into a commercially available limb extension. In their case series of 10 patients, the authors demonstrated the feasibility of physician-modified endograft–IBD creation, achieving 100% technical success and no branch occlusions at 12-month follow-up.

This design aims to facilitate catheterization of the HA in small CIA diameters, which may be hindered when using external branches due to incomplete branch opening in restricted spaces. Unfortunately, the diameters at the iliac bifurcation are not reported in this study. This information is particularly important, as small iliac bifurcation diameters represent one of the most significant—if not the primary—limiting factors for the use of currently available IBDs in real-life scenarios and therefore in this context this new concept might prove especially appealing.

Regarding the hemodynamic behavior of this novel device, it should be considered that in iliac diameters ≥16 mm, a 16-mm limb would be expected to fully expand, resulting in minimal space competition between the HA and CIA segments. However, when deployed in smaller diameters, compression of the HA branch, CIA segment, or both may occur. Compression at the CIA level, in particular, may increase the risk of flow turbulence and subsequent thrombus formation due to the presence of a parallel stent alongside the main flow lumen, as previously demonstrated by Tricarico et al^5^ in the left subclavian artery. Such spatial constraints have been addressed in IBDs by performing angioplasty of the narrowed segments, frequently using a kissing-stent technique in which the CIA and HA segments are dilated simultaneously. Wong et al^3^ reported a series of 138 helical-design IBDs (including both helical IBD and bifurcated IBD), in which 46% of patients had an iliac bifurcation diameter <16 mm (outside IFUs) and were treated using the kissing technique, achieving excellent outcomes. As acknowledged by the authors, further investigations incorporating computational fluid dynamics could help elucidate these hemodynamic effects, particularly in anatomies with limited available space.

We believe that another important technical consideration is the use of self-expandable stents as bridging stents, not only to accommodate complex anatomy but also to better withstand external compression without permanent deformation, in contrast to balloon-expandable stents. This characteristic is particularly relevant when future interventions requiring large-bore sheaths are anticipated, as the inner branch within a reduced CIA diameter may be compressed. If bridged with a balloon-expandable stent, such compression could lead to irreversible collapse and subsequent occlusion.

This study presents a promising option for East Asian populations by addressing an unmet need for more anatomically tailored devices, in this case IBDs. Although the reported results are certainly exceptional, they are based on a small patient cohort and a limited follow-up period. Therefore, more robust clinical data—including larger patient numbers, longer follow-up, and dedicated hemodynamic analyses—are required before extrapolating these findings to broader patient populations.

## Funding Support and Author Disclosures

Dr Kölbel has performed consulting, proctoring, and Intellectual Property, and received royalties, research, and travel grants from Cook Medical. Dr Torrealba has reported that he has no relationships relevant to the contents of this paper to disclose.
